# Is Leukocyte Telomere Length Related with Lung Cancer Risk?: A Meta-Analysis

**DOI:** 10.18869/acadpub.ibj.21.3.142

**Published:** 2017-05

**Authors:** Behrooz Karimi, Masud Yunesian, Ramin Nabizadeh, Parvin Mehdipour, Afsaneh Aghaie

**Affiliations:** 1 Department of Environmental Health Engineering, School of Public Health, Tehran University of Medical Sciences, Enghelab St., Tehran, Iran; 2 Department of Research Methodology and Data Analysis, Institute for Environmental Research (IER), Tehran University of Medical Sciences, Kargar St., Enghelab Sq., Tehran, Iran; 3 Department of Medical Genetics, School of Medicine, Tehran University of Medical Sciences, Enghelab St., Tehran, Iran; 4 High Institute for Research and Education in Transfusion Medicine, Tehran, Iran

**Keywords:** Lung cancer, Meta-analysis, Telomere length

## Abstract

**Background:**

Epidemiological studies have probed the correlation between telomere length and the risk of lung cancer, but their findings are inconsistent in this regard. The present meta-analysis study has been carried out to demonstrate the association between relative telomere length in peripheral blood leukocytes and the risk of lung cancer using an established Q-PCR technique.

**Methods:**

A systematic search was carried out using PubMed, EMBASE, and ISI before 2015. A total of 2925 cases of lung cancer and 2931 controls from 9 studies were employed to probe the relationship between lung cancer and telomere length. ORs were used at 95% CI. Random-effects models were used to investigate this relationship based on the heterogeneity test. Heterogeneity among studies was analyzed employing subgroup analysis based on type studies and the year of publication.

**Results:**

Random-effects meta-analysis revealed that patients with lung cancer were expected to have shorter telomere length than the control (1.13, 95% CI: 0.82-1.81, P=0.46). The summary of the pooled ORs of telomere length in adenocarcinoma lung cancer patients was 1 (95%CI=0.68-1.47, I^2^=93%) compared to patients with squamous cell lung cancer, which was 1.78 (95% CI=1.25-2.53, I^2^=3.9%). The meta-regression revealed that the effect of telomere length shortening, decreased and increased with the year of publication and the age of risks to lung cancer, was clearly related to short telomeres lengths.

**Conclusion:**

Lung cancer risks clearly related with short telomeres lengths. In patients with breathing problems, lung cancer risk can be predicted by telomere length adjustment with age, sex, and smoking.

## INTRODUCTION

Lung cancer is a leading cause of death worldwide. In 2008, more than 1.6 million new cases of lung cancer were diagnosed, which accounts for 13% of all new cancer cases. About 1.4 million people have lost their lives due to lung cancer that is estimated to be responsible for 18% of all deaths from lung cancer[1,2]. Two basic histologic subgroups of lung cancer include small cell lung cancer and non-small cell lung cancer. The non-small cell lung cancer is divided into two subtypes containing adenocarcinoma and squamous cell carcinoma (SCC)[3].

Telomeres contain up to several thousand repeated units of the sequence TTAGGG at the chromosome ends of eukaryotic cells along with associated proteins[4]. They play important roles in genomic protection and stability by preventing the destruction of nucleotides as well as hindering chromosome ends from fusion. Telomeres have an average length of 10 to 15 kb in humans and a cell growth reduction of about 50 to 200 bp per cycle[5]. Extreme shortening of telomere length leads to cellular irritation and aging and ultimately cell death[6]. Cumulative oxidative stress, chronic inflammation, and carcinogenesis happen as a result of telomere shortening in genomic DNA[7-9]. The telomere length is an indicator of the cell proliferation number during the life. Telomere length shortening is similar in different tissues, and telomere length in leukocytes is considered as a useful alternative to other tissues.

Several epidemiological studies, which are contradictory in many cases, have investigated the correlation between telomere length and various diseases, including cancers[10-15]. Some research has linked telomere length shortening to lung cancer, while others have documented a direct association between telomere length and lung cancer risk[11,16,17]. Previous studies have reported that short telomeres have an association with risks to lung cancer[11,16], whereas prospective studies have proposed that long telomeres are associated with an increased risk of lung cancer[14, 17]. Another study has also reported longer telomere length in lung cancer cases; however, other studies on telomere length in patients with lung cancer have been inconclusive and contradictory[11,18]. In the present study, a systematic meta-analysis was carried out to probe the relationship between telomere length and the risk of lung cancer as well as assess the heterogeneity among different studies.

## MATERIALS AND METHODS

### Search strategy

Following search strategies design, PubMed, EMBASE, and ISI were searched to review related articles on telomere length and the risk of lung cancer before August 2015. The search keywords included “telomere” or “lung carcinoma” or “telomere dysfunction” and “lung carcinoma” and “risk”. Abstracts and other unrelated reports were excluded from the search; only papers published in English and Persian were considered. The reference lists of each of the paper included in the study were reviewed to identify additional suitable articles. Unpublished reports were considered by sending request letters to the authors. If crucial data were not reported in the conference articles, authors were also contacted directly. Inclusion criteria included: 1) case control or cohort studies that investigated the relationship between telomere length and the risk of lung cancer; 2) sufficient information on the estimation of odd ratios at 95% CI; 3) lack of overlap between the studies in terms of the subjects.

### Study selection

The following items were extracted from each article: the information of first author, the year of publication, country, ethnicity, the number of cases and controls, the relative length of telomeres, research design, the source of DNA, and method of measuring telomere length. Literature search, study selection, and data extraction were assessed by two investigators (B.K. and M.Y.). The quality assessment and the methodological quality of studies obtained were conducted by a 13-point modified Downs and Black checklist[19] (Tables 1 and 2). In total, the checklist had 13 criteria, each containing quality concession ranging from 0 (low quality) to 1 (high quality). Uncertainties and inconsistency were resolved by consensus or discussion with another investigator.

**Table 1:** Modified Downs and Black checklist for the quality assessment of epidemiological studies

Factor	Score
**External validity**	
1. Were the subjects asked to participate in the study representative of the entire population from which they were recruited?	1
2. Were those subjects who were prepared to participate representative of the entire population from which they were recruited? Participation rate for cases and controls of at least 70%	1
Subtotal	2
**Internal validity-bias**	
3. Was an attempt made to blind those measuring the main outcomes of the exposure?	1
4. If any of the results of the study were based on “data dredging”, was this made clear?	1
5. Were the statistical tests used to assess the main outcomes appropriate?	1
6. Was compliance with the intervention/s reliable?	1
7. Were the main outcome measures used accurate (valid and reliable)?	1
Subtotal	5
**Internal validity-exposure measurement**	
8. Were measures of exposure robust? Exposure status was either documented or determined via biomarker (2); used small area ecological measures, job titles, or was self-reported (1); was based on large area ecological measures (0).	2
9. Was there a sufficient exposure gradient? The degree of variability between categories of exposure level was certain or not.	1
10. Were measures of exposure specific? Exposure measures were specific (2); based on broader, chemically-related groups (1); based on broad groupings of diverse chemical and toxicological properties (0).	2
Subtotal	5
**Internal validity-confounding**	
11. Were the cases and controls recruited from the same population?	1
12. Were the cases and controls recruited over the same period of time?	1
13. Was there adequate adjustment for confounding in the analyses from which the main findings were drawn? The study collected data on all major (2), some (including basic demographic only) (1), or no (0) potential confounders and assessed their effect in analysis.	2
Subtotal	4
Total	16

**Table 2:** Quality assessment of the included epidemiological studies

Reference	External validity		Internal validity											Total score 16

			Bias					Exposure measurement			Confounding			
			
	Item1	Item2	Item3	Item4	Item5	Item6	Item7	Item8	Item9	Item10	Item11	Item12	Item13	
[10]	1	0	0	1	1	0	1	2	1	1	1	1	1	11
[11]	1	0	0	0	1	1	0	2	1	1	1	1	2	11
[12]	1	0	1	1	1	1	1	2	1	2	1	1	1	14
[13]	1	0	0	0	1	1	1	2	1	2	1	1	2	13
[14]	1	0	0	0	1	1	1	2	0	2	1	1	2	12
[18]	1	0	0	1	1	1	0	2	1	2	1	1	2	13
[17]	1	0	0	1	1	1	1	2	1	2	1	1	2	14
[30]	1	0	0	1	1	1	0	2	1	1	1	1	2	12

The exclusion criteria included: (1) studies with no data for safety and efficacy, including protocols; (2) studies that did not report ORs or relative risk; (3) cross-sectional, review article, letters to editor, meta-analyses, case reports studies; (4) articles with the sample size less than 30 owing to insufficient information to estimate ORs.

### Data synthesis and statistical analysis

In order to simplify the analysis, the number of cases and controls were collected for both short and long telomere lengths. In these studies, data were imported into quartile 1 (shortest telomere length) and quartile 4 (highest telomere length). Q_1_, Q_2_, Q_3_, and Q_4_ were considered as short and long telomere length groups, respectively. The relationship between telomere length and the risk of lung cancer was investigated at 95% CI using ORs; thereafter, the telomere length was compared. In addition, the analysis was carried out based on the type of study (retrospective and prospective) and ethnicity (Asian or European, United States).

To assess heterogeneity among studies, the Q-test was used and the value of *P*<0.10 was considered significant. The amount of heterogeneity was also calculated by measuring the I^2^ statistic. If the value of I^2^ is equal to 0, 25, and 75%, it indicates the lack of heterogeneity, less heterogeneity, and high heterogeneity in the studies, respectively[20]. Random-effects model[21] and fixed-effects model[22] were employed to measure the effect and the size relationship of the various studies based on the Mantel-Haenszel and DerSimonian and Laird methods[21], respectively. When *P*≥0.10, the fixed-effects model was used, and when I^2^>50% and *P*<0.10, the random-effects model was used[22,23]. For each study, the OR was drawn using forest plots as well as symbols whose size corresponds to the effect and size of the study[24].

The publication bias potential was studied using a funnel plot, in which effect-sizes was plotted against the SE. Begg’s test was used to measure the asymmetry, and the value of *P*<0.10 was considered significant[25,26]. The number of missing studies due to publication bias was approximated by the trim and fill method[27]. The source of heterogeneity was analyzed using meta-regression. According to the previous information, the average age of the participants, pack-years, latitude and year of study were analyzed. In addition, the subgroup analysis was conducted based on year of study, smoking status, type of studies and ethnicity to obtain the source of heterogeneity in the studies. Sensitivity analysis was conducted to determine the effect of exclusion of any study on the change of ORs in all studies[28]. All statistical analyzes were performed using R3.0.1[29] and SDATA12.

## RESULTS

### Search results and study characteristics

**Fig. 1:**
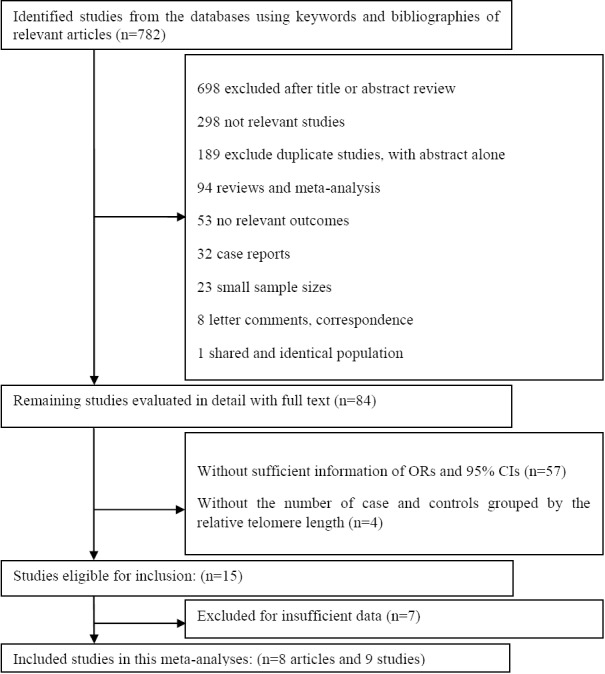
Flowchart of literature selection and study identification

A total of 782 articles were identified in the present systematic review. After reviewing the titles and abstracts, 698 articles were removed. The texts of the remaining 84 articles were read for thorough review, and after a qualitative assessment, 71 articles were also excluded from the review process. Finally, 9 studies with 2925 cases and 2931 controls met the inclusion criteria. Other details about the selection of articles are presented in Fig 1.

Among the articles studied, four originated from the United States, two from China, one from Korea, and one from Southwest Finland. Overall, a total of six studies used as controls were obtained from population-based studies and two from hospital-based studies. The most important specifications of the included studies are presented in Table 3.

**Table 3:** Specifications of included studies in the current meta-analysis

First author, year, study period	Country	Study design	Sample size ca/co	Age, males, and females	Telomere length (T/S ratio or TL)	Measurement method of telomere length	Cell type	Ref.
Sanchez-Espiridion (2014) September 1995 to March 2010	USA	Case/control	1385/1385	62.49±10.3	1.23±0.38	Q-PCR	PBLs	[18]
				62.38±10.3	1.14±0.37			
				65.00±8.7	1.10±0.44			
				64.82±8.6	1.13±0.33			
Jang (2008) January 2005 and July 2005	Korea	Case/control	243/243	59.2±6.6	1.59±0.75	Q-PCR	PBLs	[11]
				59 ±6.5	2.16±1.10			
Seow (2014)	USA	Prospective cohorts	847/847	61.37±6.53	1.21±0.43	Q-PCR	PBLs	[17]
				61.12±6.35	1.18±0.39			
Sun (2015) 1998-2004	USA	Case/control	191/207	67±12.2	98 101	Q-FISH	Blood lymphocyte	[30]
				66.3±12.1				
Wu (2003)	USA	Case/control	54/54	64.7 ±8.9	1.1 1.4	Q-PCR	PBLs	[10]
				64.5±9.0				
Hosgood III (2009) March 1995 to March 1996	China	Case/control	120/110	55 ±11.9	NA	Q-PCR	Sputum samples	[12]
				54.7 ±13.5				
Shen (2011) 1985-1988	Finland	Case/control	229/229	59±5	1.14 (0.23) 1.10 (0.22)	Q-PCR	PBLs	[13]
				58±5				
Lan (2013) 1997 to 2000	China	Nested case-control	215/215	40-65	1.37–1.60	Q-PCR	Blood sample	[14]

PBLs, peripheral blood leukocytes; Q-FISH, Quantitative Fluorescent in situ hybridization; Q-PCR, real-time polymerase chain reaction; Ref., reference, TL., telomeres length

### Meta-analysis results

The total of all eligible studies in the meta-analysis showed that there is a relationship between the increased risk of lung cancer and the telomere length shortening 1.13 (0.82–1.81, *P*=0.46).

**Fig. 2:**
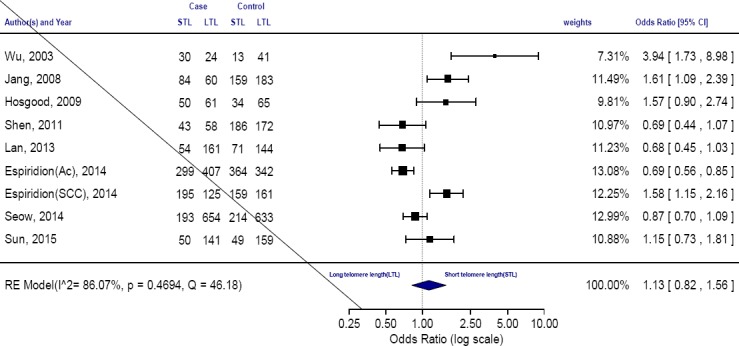
The forest plot comparing the association between telomere length and the risk of lung cancer (random-effect model). STL, short telomeres length; LTL, long telomeres length

The rate of heterogeneity was 86% (I^2^=86.07%, *P*=0.0469, Q=46.1817) in the present study, which indicated a high level of heterogeneity. Under these conditions, the amount of pooled ORs was determined using the Random-effects model. Pooled ORs studies revealed that telomere length shortening increases the risk of lung cancer (OR=1.13, 95% CI: 0.82–1.81) (Fig. 2). The random-effects model indicated that there are significant differences between the subgroup analyses.

A subgroup analyses were performed in order to discover the source of heterogeneity and further explore the effects of the histologic subtype of lung cancer on telomere length as well as evaluate any differences between trials by ruling out the confounding effect of the histologic subtype of lung cancer. The pooled summary of ORs of telomere length in adenocarcinoma lung cancer patients was 1 [95% CI=0.68-1.47, I^2^=93%] compared to 1.78 [95% CI=1.25-2.53, I^2^=3.9%] for SCC lung cancer. The pooled ORs showed that patients with SCC lung cancer were expected to have shorter telomere length than the control; however, it was found that there was no relationship between telomere length and the risk of lung cancer in patients with adenocarcinoma lung cancer. Overall, pooled ORs for both subtype was 1.40 (95% CI: 0.87–2.27, I^2^=90%, *P*=0.16612), which indicated shorter telomere length in both lung cancer subtype.

**Fig. 3:**
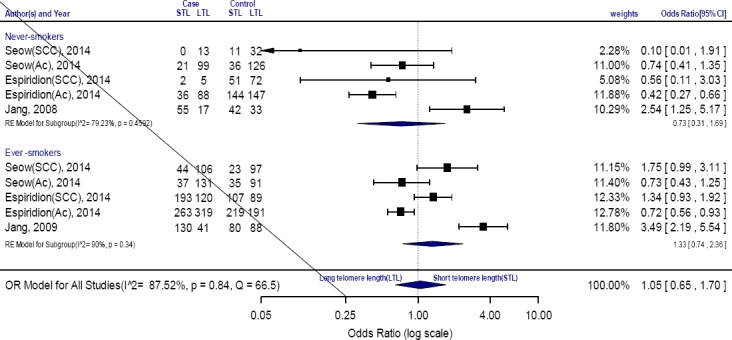
Forest plot of the subgroup analyses stratified by smoking status. STL, short telomeres length; LTL, Long telomeres length

Sub-analysis based on the smoking status was conducted to verify the heterogeneity. It seemed that there may exist a marginal heterogeneity in the ever smokers (*P*=0.34, I^2^=90%). We performed a subgroup analysis for association between telomere length shortening and lung cancer based on the smoking status. The OR for association between telomere length shortening and lung cancer in <30 pack-years subgroup is 1.1 (0.4–3.01, I^2^ 87.4%) and in ≥30 pack year is 1.51 (0.65–3.5, I^2^ 88.8%). The pooled results based on all included studies for smoking status indicated a remarkable relation among the lung cancer risk and telomere length shortening 1.05 [0.65–1.7, I^2^= 89.60%] (Fig. 3) and for cumulative smoking 1.29 [95% CI=0.71–2.35, I^2^=87%]. A direct association of telomere length shortening with the risk of lung cancer was found for males [1.43 (0.81–2.53), I^2^ 87 %, *P*=0.1], and females [1.06 (0.58–1.73), I^2^ 79%, *P*=0.8] and age 1.32 [95% CI=0.52–1.95, I^2^ = 93.58%] (Fig. 3).

**Fig. 4:**
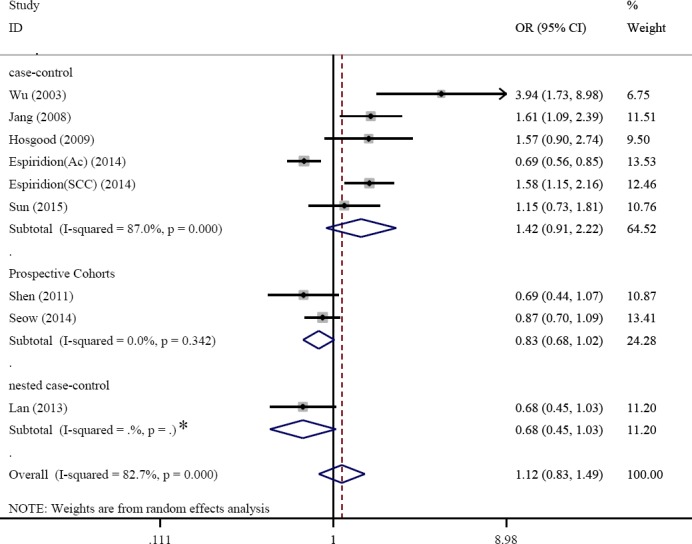
Forest plot of the subgroup analyses stratified by type of studies (case- control, nested case- control and prospective cohorts). *One study selected, therefore P-values wasn’t calculated.

**Fig. 5:**
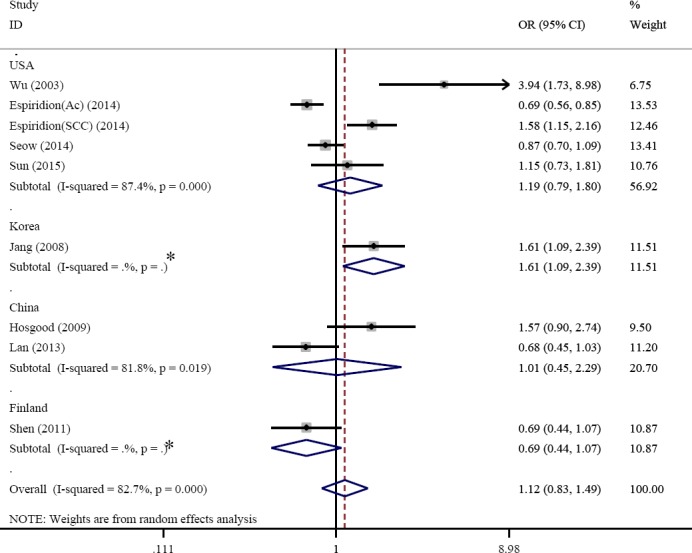
Forest plot of the subgroup analyses stratified by country (USA, Korea, China and Finland). *One study selected, therefore P-values wasn’t calculated.

Considering the availability of only six retrospective and three prospective studies, the heterogeneity was assessed based on these studies. The results of the retrospective studies demonstrated a significant relationship between the telomere length shortening and the increased risk of lung cancer; however, there was no significant correlation was found from the prospective studies (Fig. 4). The ethnicity-based classification also showed a significant relationship in this regard. The relationship between the study type and telomere length and the risk of lung cancer was also investigated. The results displayed a correlation between telomere length shortening and the risk of lung cancer obtained in previous studies conducted in United States[10,17,18,30]. However, such correlation was observed in studies conducted in Asian[11,12] and European[13] countries (Fig. 5). To investigate other causes of heterogeneity among the studies, the meta-regression model was employed, in which *P* values were significant for latitude study, smoking, and the average age of the participants, but there was no significant correlation for the year of study. It was also found that the year of study and the average age of the participants could be the reason for the heterogeneity among the studies. However, the effect of other parameters such as latitude and average smoking cannot be a cogent reason for the heterogeneity among the studies.

### Publication bias analysis

**Fig. 6:**
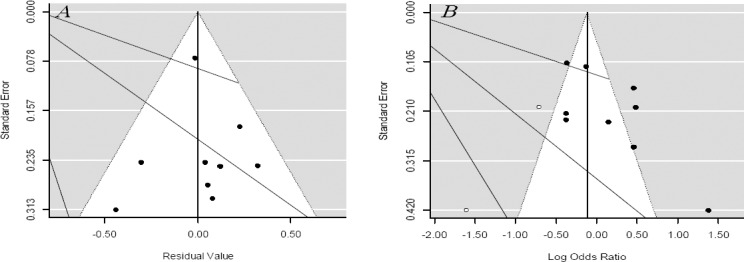
Begg’s funnel plot for publication bias test. Each point represents a separate study for the indicated association. (A) Funnel plot for mixed effects model of telomere length and the risk of lung cancer adjusted for age and smoking; (B) Funnel plot adjusted with trim and fill method examining the publication bias of telomere length and the risk of lung cancer. Each point represents a separate study for the indicated association. Circles black, included studies; Circles blank, presumed missing studies

The validity of the meta-analysis results was assessed by publication bias estimation. The shape of the funnel plot was slightly asymmetrical (Fig. 6a), and the Egger’s regression (funnel plot asymmetry test) intercept were t=2.63 and *P*=0.039, suggesting that there was possible publication bias among the nine studies. For evaluation of asymmetry in the funnel plot, hypothetically non-published studies were found by trim and fill method, which was computed for two potentially missing studies due to the publication bias (Q=69.32, *P*=0.03) (Fig. 6b).

**Fig. 7:**
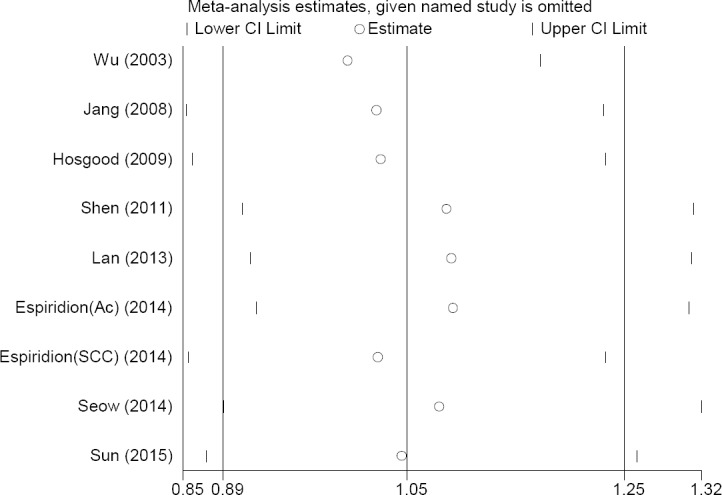
Sensitivity analysis for all studies. The point estimates of sensitivity for each study are shown as circles. Error bars are 95% CI.

Sensitivity of these nine studies was demonstrated by forest plots as shown in Figure 7. The results demonstrated that the pooled sensitivity and specificity of the studies were 0.48(0.2–0.68) and 0.43(0.18–0.57), respectively. The positive likelihood ratio, diagnostic OR, negative likelihood ratio, I^2^ of sensitivity and specificity were 84.9% (*P*=0.001), 71.7% (*P*=0.001), 67.9% (*P*=0.001), 59.1% (*P*=0.008), and 81.1% (*P*=0.001), respectively. There was also little difference in the sensitivity analysis due to inaccuracies in the data presented or incorrect information in the articles.

## DISCUSSION

To investigate the relationship between lung cancer and telomere length shortening in this meta-analysis, 2925 lung cancer cases and 2931 controls of 9 different articles were used (OR=1.13). The measurement method for the telomere length in all included studies, except Sun *et al*.[30] was real-time PCR, which was not very effective in obtaining the results of the meta-analysis.

The greater number of studies showed significant associations between shorter telomere lengths in peripheral blood leukocytes and the risks of different subtype of lung carcinomas. Wu *et al*.[10] and Jang *et al*.[11] found significantly a raised risk of lung cancer related to shortened telomere length (4.08–18.71, OR=8.73). Conversely, Lan *et al*.[14] indicated that the longer telomere length in peripheral blood cells was positively and remarkably related to the risk of lung cancer (OR=2,0.8-4, *P*=0.003)[14]. Longer telomere length may increase the risk of lung cancer by promoting immortality of the cells, which leads to abnormal rapid cell proliferation and tumor formation[31]. The results showed that there was a significant correlation between telomere length shortening and the risk of lung cancer. Studies have shown that telomere length is very important to human life and plays an essential role in maintaining genomic integrity. Any disruption in telomere lengths may result in genetic changes and eventual formation of malignant tumours[6,7]. In addition, disruption of the telomere is associated with a reduction in DNA repair capacity and cytogenetic abnormalities[31]. Both animal studies and clinical observations have demonstrated that telomere length shortening is associated with an increased risk of cancer, such as epithelial cancer. However, telomere length shortening plays a contradictory role in the development of lung cancer. The progressive loss of telomeric repeated unit in each cell division leads to aging and limited cellular proliferation capacity[32]. Consequently, telomere length shortening leads to prevention of the activities of other tumor suppressor genes[31]. When the telomeres reach a critical length, a chromosome may break, resulting in increased genomic instability and malignant transformation potential through fusion-bridge-breakage cycles[7]. Furthermore, a consequence of their high guanine content is that they are sensitive to damage from chronic inflammation and cumulative oxidative stress, which results in telomeric single-strand breaks and the loss of distal telomere parts[32].

Various case control studies have reported shorter telomeres to be associated with the risk of lung cancer, whereas results from Seow *et al*.[17] showed the opposite effect[17]. They demonstrated that the effect of long telomere length on risk of lung cancer was especially for adenocarcinoma, and specifically among females[17]. Sanchez-Espiridion *et al*.[18] found a differential relationship between telomere length and the risk of adenocarcinoma and SCC, in which long telomeres length was associated with the risk of adenocarcinoma but reduced risk of SCC risk[18]. Adenocarcinoma is more commonly found among females than men and is the most common lung cancer subtype among lifelong non-smokers.

Telomere shortening has also been associated with smoking status[13]. Our meta-analysis indicated a significant increase in lung cancer and smoking status (OR ever smoker=1.33 and OR heavy smoker (pack years ≥31-years) =1.51). The study has been approved by other researchers. Valdes *et al*.[33] demonstrated that telomere length shortening is highly affected by smoking, which is a confounding factor and effect-measure modifier. Several possible mechanisms can describe these effects[13]. Smoking is known to be related to systemic inflammation and oxidative stress[33,34]. The high destruction of telomere length in smokers is probably a part of the oxidant-induced senescence phenomenon[13]. Jang *et al*.[11] reported that the risk of lung cancer was related to short telomere length in both younger people and those above 61 years; however, there was a stronger relationship in the younger age population[35]. Shen *et al*.[13] observed longer telomere length only in male smokers above 38 years old; however, in males younger than 38, longer telomere was not observed. Results of the current meta-analysis indicated that smoking is remarkably related to shortening of telomeres in peripheral blood leukocytes. Also, older age is positively related with telomere shortening (OR=1.32)[33].

In conclusion, a positive association was found between short telomere length and the risk of lung cancer. In patients with breathing problems, the risk of lung cancer can be predicted by telomere length adjusted with age, sex, and smoking. The molecular mechanisms that induce a decrease in telomere length may be implicated in the expansion of histologic subtypes of lung cancer. The several remarkable advantages of the current study include the fact that search strategy was employed extensively with reviewing of titles and abstracts manually. The search period spanned for a long time so that studies are not excluded based on the years of publication. Finally, further large studies will be required to authenticate these results and evaluate the effect of genetic and environmental risk factors before and after cancer diagnosis.
